# Regulation of Yeast Cytokinesis by Calcium

**DOI:** 10.3390/jof11040278

**Published:** 2025-04-02

**Authors:** Qian Chen

**Affiliations:** Department of Biological Sciences, The University of Toledo, 2801 West Bancroft Street, Toledo, OH 43606, USA; qian.chen3@utoledo.edu

**Keywords:** calcium, Pkd2, *Schizosaccharomyces pombe*, fission yeast, cytokinesis

## Abstract

The role of calcium, an essential secondary messenger, in cell division remains an outstanding question in cell biology despite several significant findings over the past few decades. Among them is the landmark discovery of intracellular calcium waves during cytokinesis, the last stage of cell division, in fish cells. Nevertheless, subsequent studies have been largely unable to determine the underlying molecular mechanism of these cytokinetic transients. At the center of this stalemate stands two challenging questions, how these calcium transients rise and what they do during cytokinesis. Yeast, despite its proven prowess as a model organism to study cell cycle, has not drawn much interest in addressing these questions. However, the recent discovery of cytokinetic calcium spikes in the fission yeast *Schizosaccharomyces pombe* has provided novel insights into how calcium regulates cytokinesis. In this review, I will primarily focus on our current understanding of the molecular mechanism of cytokinetic calcium transients in yeast cells. First, I will briefly recount the discovery of cytokinetic calcium transients in animal cells. This will be followed by an introduction to the intracellular calcium homeostasis. Next, I will discuss yeast cytokinetic calcium spikes, the ion channel Pkd2 that promotes these spikes, and the potential molecular targets of these spikes. I will also compare the calcium regulation of cytokinesis between yeast and animal cells. I will conclude by presenting a few critical questions in our continued quest to understand how calcium regulates cytokinesis.

## 1. Introduction

Almost thirty years have passed since the landmark discovery of cytokinetic calcium (Ca^2+^) waves in Japanese rice fish (*Oryzias latipes*) cells [1], but the underlying molecular mechanism remains very much unknown. It has long been known that Ca^2+^ is essential to cytokinesis [2]. However, the question of why was never answered. By injecting a fluorescent Ca^2+^ reporter protein aequorin into the fish eggs [1], the group of Lionel Jaffe revealed for the first time that the Ca^2+^ level increases dramatically in a few seconds at the ingressing cleavage furrow. Such Ca^2+^ transients then quickly spread towards the poles of the dividing eggs. Therefore, the authors named them Ca^2+^ waves. This is the first demonstration that the last stage of cell division cytokinesis is concurrent with the rise and fall of intracellular Ca^2+^ transients. Subsequent studies by many other groups confirmed the existence of similar cytokinetic Ca^2+^ transients in either the oocytes or eggs of animals including zebrafish (*Danio rerio*) and African clawed frog (*Xenopus laevis*) [3,4] (for a review of calcium transients in animal cells, see [5]). The discovery of these Ca^2+^ transients firmly established a link between Ca^2+^ and cytokinesis in animal eggs and embryos.

These earlier discoveries generated both excitement and many unanswered questions. The chief ones are what role these Ca^2+^ waves play in cytokinesis and how they are regulated. Between the two, the first question appears easier to answer. Two groups, including that of Lionel Jaffe, worked independently by injecting Ca^2+^ chelators into dividing cells [3,6]. They found that both cleavage furrow ingression and the following cell separation are inhibited by the chelators. This would have settled the first question. However, another research group, led by Issei Mabuchi, made a starkly opposite finding that the Ca^2+^ chelators had no effect on either the cleavage furrow ingression or the daughter cell separation of frog eggs [7]. They concluded that cytokinetic Ca^2+^ waves have no significant role in cytokinesis. These contradicting results raised the question of whether there is a causal relationship between Ca^2+^ waves and cytokinesis. This controversy likely dented enthusiasm towards the study of these cytokinetic Ca^2+^ waves, partially contributing to a prolonged stalemate in determining their molecular mechanisms. Therefore, three decades after the discovery of these Ca^2+^ transients, their origin and cellular function have remained largely unknown.

The interest in this decades-old question is rekindled by the recent surprising discovery of cytokinetic Ca^2+^ spikes in the unicellular model organism *S. pombe*. In this review, I will focus on what we have learned about the regulation of fission yeast cytokinesis by Ca^2+^. I will start by summarizing our current understanding of the molecular mechanism of cytokinesis and Ca^2+^ homeostasis. This will be followed by a discussion of the newly discovered cytokinetic Ca^2+^ spikes. I will then review the findings demonstrating how a putative mechanosensitive cation channel Pkd2 promotes Ca^2+^ spikes in cytokinesis. Then, I will discuss the potential targets of these spikes including the essential Ca^2+^-binding molecules calmodulin and calcineurin. Lastly, I will discuss the challenges and the future directions for us to fully understand the role of Ca^2+^ in cytokinesis.

## 2. Molecular Mechanism of Cytokinesis

Cytokinesis is the last stage of cell division. During this stage, the cytoplasm of daughter cells separate from each other, following the separation of chromosomes during mitosis. Cytokinesis failure can lead to polyploidy and is associated with many human diseases including cancers [8]. As an essential cellular process, cytokinesis has been extensively studied since the early days of cell biology. In particular, over the last three decades, we have made substantial advances in understanding the molecular mechanism of cytokinesis. These studies have revealed that cytokinesis is highly conserved in almost all eukaryotic organisms including yeast, social amoebae, and animals (for a review, see [9]).

Like many eukaryotic cells, fission yeast cytokinesis can be divided into four steps (for a review, see [10]). The first is the selection of the equatorial division plane, a process largely depending on the nucleus [11]. This is followed by the assembly of the actomyosin contractile ring at the division plane. This actin-centric process requires many actin cytoskeletal proteins including the motor protein type II myosins, the actin nucleator formin, and the actin filament-severing protein cofilin [12,13,14]. The third step is the ingression of the cleavage furrow, driven by mechanical force, jointly produced by the contractile ring, the septum, and turgor pressure [15]. The last step is the separation of daughter cells. Fission yeast cell separation relies on the assembly of the septum, a process regulated by the septation initiation network (SIN) pathway (for a review, see [16]).

The primary advantages of using fission yeast to study the molecular mechanism of cytokinesis are two. First, yeast genetics is a powerful tool to study cytokinesis. It has helped to discover many essential genes required for cytokinesis (for a review, see [16]). Most of these genes are conserved in animal cells. The second advantage is the application of quantitative microscopy. The method, not yet available in most other model organisms, has allowed precise measurement of molecular number of proteins in live fission yeast cells [17]. Better yet, it can be combined with computer-assisted image analysis to quantify thousands of cells all at once [18]. However, compared to other model organisms such as *C. elegans* (round worm) and *D. melanogaster* (fruit fly), the method of Ca^2+^ imaging remains under-developed in yeast. Despite our extensive understanding of the mechanism of cytokinesis, many questions regarding this essential cellular process remain open [19]. Among them, one of the most important ones is the role of Ca^2+^ in cytokinesis.

## 3. Ca^2+^ Homeostasis of Eukaryotic Cells

Ca^2+^ is one of the most important secondary messengers in living organisms. Due to the importance of Ca^2+^ in signaling, the intracellular [Ca^2+^] of eukaryotic cells is tightly regulated. In the cytoplasm, [Ca^2+^] remains relatively low, in the nanomolar range. In comparison, several intracellular organelles including the endoplasmic reticulum (ER) and lysosomes maintain much higher [Ca^2+^]. Extracellular [Ca^2+^] is also higher, in the micromolar range. As a result of this asymmetric distribution of Ca^2+^, a transient increase in cytoplasmic Ca^2+^ level will trigger downstream signaling pathways in diverse cellular processes, including exocytosis, muscle contractility, and neurotransmission.

Like animal cells, yeast cells keep their cytoplasmic [Ca^2+^] much lower than that of the extracellular environment. The [Ca^2+^] in the fission yeast cytoplasm is about 100 nM [20]. Like most eukaryotic cells, yeast maintains much higher [Ca^2+^] in two intracellular organelles, the ER and the lysosome equivalent of vacuoles. In budding yeast cells, the ER [Ca^2+^] is ~10 µM, and the vacuolar [Ca^2+^] is ~2 mM [21].

A large number of Ca^2+^ channels, transporters, and pumps help maintain the homeostasis of Ca^2+^ in a eukaryotic cell (for a review, see [22]). The channels permeate Ca^2+^ in the direction of the ion gradient. It does not require any energy. A well-studied example are the voltage-gated Ca^2+^ channels (VGCCs) found in animal cells (for a review, see [23]). Fission yeast possesses only one putative VGCC [24]. Cch1 is the presumed α-subunit of this channel and Yam8 is projected to be the β-subunit [25]. Compared to a channel, a transporter couples the flow of Ca^2+^ to that of another ion. For example, Ca^2+^-sodium exchangers (NCXs) remove Ca^2+^ from the cytoplasm by coupling it to the flow of Na^+^. Budding yeast possesses one putative NCX in Ycx1 [26], but no such transporter has been identified yet in fission yeast. Lastly, a Ca^2+^-ATPase moves Ca^2+^ against the ion gradient by utilizing the energy produced by ATP hydrolysis. Fission yeast possesses several Ca^2+^-ATPases in intracellular organelles including the ER-specific Pmr1 [27] and Cta4 [28], the vacuolar pump Pmc1 [27], and the Golgi-localized Cta5 [29]. Overall, yeast cells maintain similar intracellular Ca^2+^ homeostasis to that of animal cells.

## 4. Cytokinetic Ca^2+^ Spikes in Yeast

Similar to animal cells, yeast requires Ca^2+^ for cell division [30], but the existence of cytokinetic Ca^2+^ transients in yeast was not known until recently. In the past, organic Ca^2+^-sensitive dyes, such as Fura-2 developed by the group of Roger Tsien, were commonly used to detect Ca^2+^ in live cells [31]. However, these dyes do not usually penetrate either the cell wall or the plasma membrane of yeast efficiently. Injection, a commonly used strategy in large animal cells, unfortunately is not applicable to yeast. The technical advance that helped uncover cytokinetic Ca^2+^ transients in fission yeast came from an unexpected source, the study of neurons. Over the last decade, genetically encoded Ca^2+^ indicators, in particular GCaMP [32], have revolutionized the study of Ca^2+^ transients by neuroscientists. This reporter consists of the permuted Green Fluorescence Protein (GFP), calmodulin, and the M13 peptide from myosin light chain kinase (MLCK) [33]. It has been widely used to detect Ca^2+^ transients among excitable neuronal cells. The newest edition jGCaMP8 has even achieved temporal precision and sensitivity, comparable to the traditional electrophysical recording in neuronal cells [34]. However, its application in the detection of Ca^2+^ transients in non-excitable cells, particularly in yeast, remains limited.

To investigate Ca^2+^ transients in fission yeast cells, our lab has developed a novel Ca^2+^ imaging approach by combining GCaMP with quantitative microscopy. Poddar et al. first integrated the GCaMP coding sequence into the genome of fission yeast [35]. This reporter detects the Ca^2+^ transients triggered by sudden downshift of extracellular osmolarity. To our knowledge, this is the first demonstration of intracellular Ca^2+^ transients in this widely used unicellular model organism. To determine whether intracellular [Ca^2+^] rises during cytokinesis, Poddar et al. took two complementary approaches. First, they examined the correlation between intracellular Ca^2+^ and cell-cycle stages in an asynchronized population. Secondly, they employed time-lapse fluorescence microscopy to measure [Ca^2+^] in the dividing cells. Both methods detect a dramatic increase in intracellular [Ca^2+^] during cell division.

**Figure 1:**
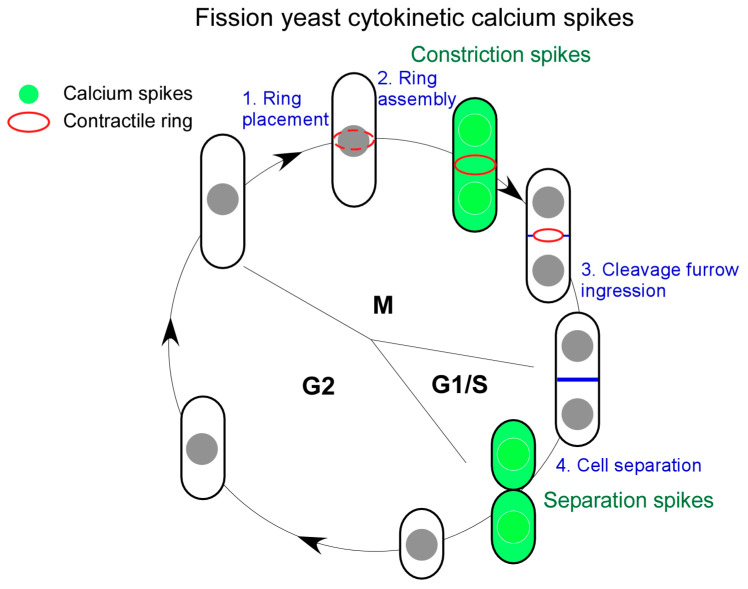
Cytokinetic Ca^2+^ spikes of fission yeast cells. Fission yeast cytokinesis, in four consecutive steps (blue), is accompanied by two distinct Ca^2+^ spikes (green) during both M and G1/S phases of cell cycle. “Constriction spikes” rise at the start of the contractile ring constriction. In contrast, “Separation spikes” appear at the end of cytokinesis when two daughter cells separate.

Two Ca^2+^ spikes rise during fission yeast cytokinesis (Figure 1). They are named “Constriction spikes” and “Separation spikes”, respectively. As the name implies, the former accompanies cleavage furrow ingression and the concurrent contractile ring constriction. Interestingly, this wave of Ca^2+^ spikes remains active throughout the duration of cleavage furrow ingression for ~30 min. This is followed by the second Ca^2+^ spikes during daughter cell separation (Figure 1). Surprisingly, in ~40% of dividing cells, the spikes rise asymmetrically, appearing in two daughter cells sequentially. The asymmetry of fission yeast cytokinesis [36] may have played a role in this asymmetry of Separation spikes.

## 5. Regulatory Mechanism of Cytokinetic Ca^2+^ Spikes

The discovery of cytokinetic Ca^2+^ spikes in fission yeast cells leads to a similar question raised by the study of Fluck et al. [1] more than three decades ago, where Ca^2+^ comes from. There are two likely sources, one through the influx of Ca^2+^ from the extracellular environment and the other through efflux from the intracellular organelles. Both may have contributed to the cytokinetic Ca^2+^ waves of animal cells [5,37].

**Figure 2:**
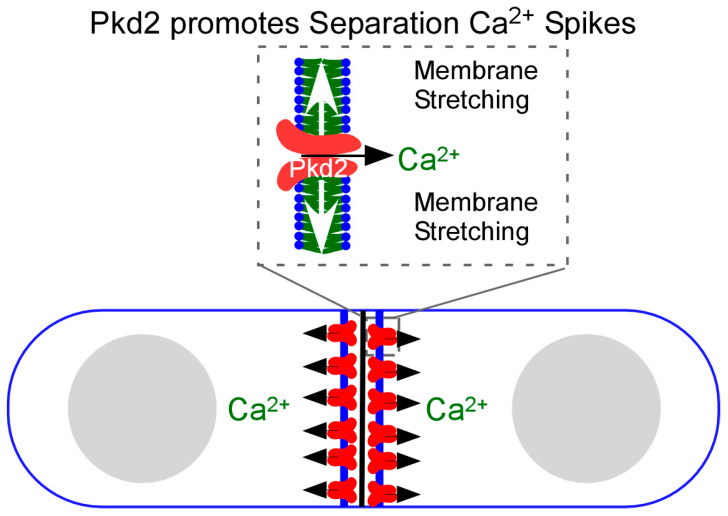
Pkd2 promotes Ca^2+^ influx during the daughter cell separation. Pkd2 is recruited to the plasma membrane at the equatorial division plane during cytokinesis. The membrane expansion during the cell separation is presumed to activate this mechanosensitive channel through stretching the plasma membrane at the division plane. This channel then promotes Ca^2+^ influx, leading to the Separation spikes.

Surprisingly, fission yeast cells employ a putative force-sensitive ion channel Pkd2 to promote Ca^2+^ influx during cytokinesis (Figure 2). Pkd2 is the ancient homologue of the animal polycystin channel [38]. Two such channels, PC1 and PC2, can be found in animal cells, including humans. Mutations of human polycystin genes, *Pkd1* or *Pkd2*, lead to one of the most common human genetic disorders, Autosomal Polycystic Kidney Disorder (ADPKD) (for reviews, see [39,40]). Fission yeast Pkd2 is an essential protein localized on the plasma membrane throughout the cell cycle [41,42,43]. During interphase, Pkd2 is enriched at the cell tips and the plasma membrane invagination sites called eisosomes [44]. It is frequently internalized from the plasma membrane through the endocytic pathway, depending on its C-terminal cytoplasmic tail, like many G-protein-coupled receptors [44]. Pkd2 regulates the fission yeast turgor pressure required for the cell size expansion during interphase [45]. During cytokinesis, this putative channel is recruited to the equatorial division plane at the time of cleavage furrow ingression.

Fission yeast Pkd2 is essential for the last step of cytokinesis, daughter cell separation. Its depletion leads to much slower separation of daughter cells or a complete failure. Fittingly, Pkd2 contributes significantly to the Separation Ca^2+^ spikes [46]. Without it, the second wave of cytokinetic Ca^2+^ transients largely disappears. This ion channel is likely activated through the plasma membrane stretching produced by the cytokinetic machinery (Figure 2). Membrane stretching activates the in vitro reconstituted Pkd2 in giant unilamellar vesicles (GUVs) to allow the passage of Ca^2+^ through this channel. To our knowledge, Pkd2 is the only known yeast Ca^2+^ channel that contributes to cytokinetic Ca^2+^ spikes thus far.

In addition to Pkd2, fission yeast possesses other mechanosensitive ion channels that may contribute to cytokinetic Ca^2+^ spikes. Msy1 and Msy2 are the homologues of the bacterial MscS channel [47]. They provide the essential Ca^2+^ spikes required for adaption to hypo-osmotic shocks of yeast cells [48]. These transients promote lipid transfer from the ER to the plasma membrane, preventing potential membrane rupture [49]. Trp1322 and Trp633 are another two potential mechanosensitive channels [50]. Their predicted tertiary structures are similar to that of Pkd2, but they localize to the plasma membrane and the intracellular membrane structures, respectively [44]. The function of these mechanosensitive channels in cytokinesis remains to be elucidated.

## 6. The Effectors of Cytokinetic Ca^2+^ Spikes

The function of cytokinetic Ca^2+^ transients has long been debated. On one side, many researchers [1] argued that these intracellular Ca^2+^ transients promote cleavage furrow ingression. The transients could activate contraction of the actomyosin contractile ring and promote membrane trafficking at the cleavage furrow [5]. This hypothesis is based on the observations that chelation of intracellular Ca^2+^ during cytokinesis inhibits cleavage furrow ingression [3,6,51]. Such a model fits the long-standing theory that the constriction of cytokinetic contractile ring, like muscle contraction, is triggered by Ca^2+^. In skeletal muscle cells, Ca^2+^ activates the type II muscle myosin through myosin light chain kinase (MLCK) and troponin to initiate actomyosin contractility (for a review, see [52]). However, this model has been controversial [7]. To identify the effects of cytokinetic Ca^2+^ transients, an approach combining quantitative cell biology and genetics is direly needed.

Fission yeast provides a strong platform for analyzing the importance of Ca^2+^ transients in cytokinesis quantitatively. Both the temporal and spatial regulation of fission yeast cytokinesis have been carefully measured [17]. The molecular numbers of many components of the cytokinetic machinery, including actin, myosin II, and formins, have been measured through the pioneering method of quantitative microscopy [18,53]. Its cytokinesis has even been simulated in silico using a computation model, the first among all model organisms [54]. Taking advantage of these quantitative tools, Poddar et al. identified two critical roles of cytokinetic Ca^2+^ spikes. One is to promote cleavage furrow ingression and the other is to safeguard daughter integrity during cell separation [35]. The study of fission yeast thus demonstrates clearly that these transients are crucial for both temporal regulation and the robustness of cytokinesis.

**Figure 3:**
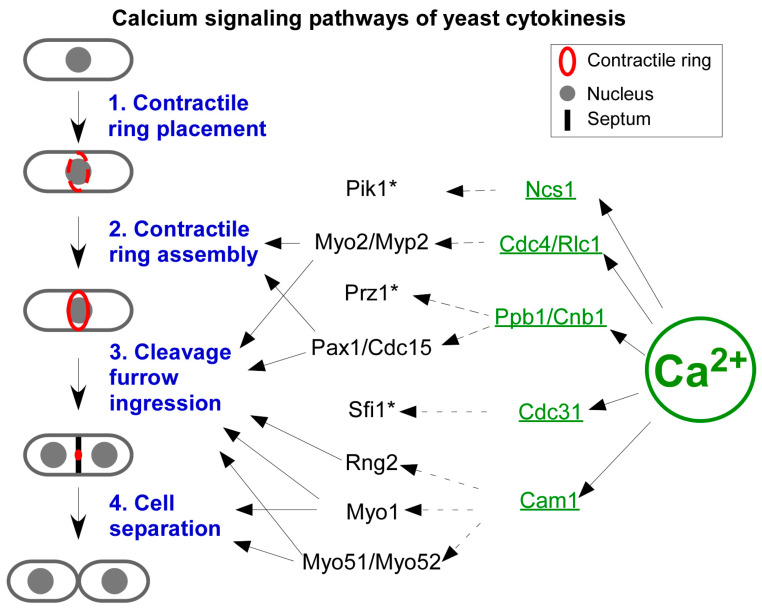
A model of the Ca^2+^ signaling pathways in fission yeast cytokinesis. Ca^2+^ directly binds and activates five EF-hand proteins (green) including Ncs1, the type II myosin light chains Cdc4 and Rlc1, the calcineurin Ppb1 and Cnb1, the centrin Cdc31, and the calmodulin Cam1. By interacting with a wide range of targets, these Ca^2+^ sensors promote the last three steps (blue) of cytokinesis, including contractile ring assembly, cleavage furrow ingression, which is coupled to contractile ring constriction, and cell separation, which is concurrent with septation. *: These proteins have no clear role in cytokinesis.

Yeast genetics provide another powerful tool in determining the potential targets of cytokinetic Ca^2+^ spikes. Decades of genetic studies have generated hundreds of either deletion or conditional mutants of the genes that are important to cytokinesis. A deletion library of more than 3000 non-essential fission yeast genes is also available commercially [55]. All these mutants can be leveraged to identify the molecules activated by a sudden increase in [Ca^2+^] either directly or indirectly during cytokinesis. The former includes calmodulin (CaM), calcineurin (CaN), and Ca^2+^/CaM-sensitive kinases (CaMK). The latter includes many other CaM-binding proteins including motor protein myosins and ion channels. Here, I will discuss some of the most likely targets of the cytokinetic Ca^2+^ spikes (Figure 3).

CaM is an evolutionarily conserved Ca^2+^-binding protein, found in almost all eukaryotes including fungi, amoeba, plants, and animals (for a review of budding yeast calmodulin, see [56]). Fission yeast CaM Cam1 is an essential protein [57] (Figure 3). Like other calmodulins, it contains four EF-hand motifs, each of which can bind one Ca^2+^ [58]. Cam1 requires Ca^2+^ for its essential functions [59]. This small protein distributes between two subcellular structures. First, Cam1 is a constitutive component of spindle pole bodies (SPBs) [59], the yeast equivalent of centrosomes, where it interacts with the pericentrin homologue Pcp1 [60,61]. Secondly, Cam1 is in endocytic actin patches as the regulatory light chain for the type I myosin Myo1 [62,63]. However, it remains unknown whether the interaction between Cam1 and Myo1 depends on Ca^2+^ [64]. In addition to Cam1, fission yeast also possesses a second camodulin-like protein. However, Cam2 does not bind Ca^2+^ directly [65]. Cam2 localizes to endocytic actin patches, but it is absent from SPBs. It remains unclear what the Ca^2+^-dependent functions of Cam1 are during cytokinesis.

The second likely target of Ca^2+^ spikes is calcineurin (CaN), the evolutionarily conserved Ca^2+^-, and CaM-sensitive phosphatase (for a review, see [66]). CaN consists of one regulatory and one catalytic subunit (Figure 3). The former is a CaM-like protein, whose four EF-hand motifs can each bind one Ca^2+^. The latter contains the catalytic core of this phosphatase, whose activity is auto-inhibited by its own C-terminal inhibitory domain. CaN only becomes active when it binds both Ca^2+^ and CaM. The fission yeast homologues of CaN regulatory and catalytic subunits are Cnb1 and Ppb1, respectively [67]. Neither is an essential gene, but both play critical roles in cell proliferation. During cytokinesis, Ppb1 highly concentrates at the contractile ring [68,69]. There, it interacts with two essential components of the ring, the paxillin Pxl1 and the Fes/Cip4 homology Bin/amphiphysin/Rvs (F-BAR) protein Cdc15 [68]. As a part of the contractile ring, Ppb1 promotes the proper placement of the contractile ring, the ring constriction, and the daughter cell separation [67]. One way that Ppb1 carries out its function is through dephosphorylating Cdc15 to stabilize this membrane-sculpturing protein [68]. In addition to Cdc15, Ppb1 also dephosphorylates the transcription factor Prz1 [70], whose function during cytokinesis remains unclear (Figure 3). Future work will need to determine whether any of the above-described functions of CaN depend on cytokinetic Ca^2+^ spikes.

Other potential targets of cytokinetic Ca^2+^ spikes include the PKA pathway, the centrin-like protein Cdc31, myosin light chains, the Ca^2+^ sensor Ncs1, and Ca^2+^/CaM-dependent kinases (Figure 3). Ca^2+^ regulates the association between the PKA pathway kinase Pka1 and its regulatory subunit Cgs1 [71]. Cdc31 is a CaM-like protein with four EF-hands. It is an essential component of SPBs [72]. Similarly, both the regulatory and essential light chains of yeast myosin II, Rlc1, and Cdc4, respectively, are CaM-like proteins. Both possess four EF-hands that are capable of binding Ca^2+^ directly [73,74]. Ncs1 is another CaM-like protein that binds Ca^2+^ through its EF-hands [75]. It promotes the activity of phosphatidylinositol-4 kinase Pik1 at the Golgi apparatus, similar to its budding yeast homologue Frq1 [76,77]. Like budding yeast, fission yeast also possesses two putative Ca^2+^/CaM-sensitive kinases Cmk1 and Cmk2 [78,79,80,81]. Further studies will need to determine what roles of these potential Ca^2+^ spikes’ targets play during cytokinesis.

## 7. Comparison of Ca^2+^ Homeostasis and Signaling Between Two Yeasts

Although budding yeast *S. cerevisiae* and fission yeast *S. pombe* are separated by more than 300 million years of evolution [82], these two model organisms share many similarities in their regulation of Ca^2+^ homeostasis and signaling. Both utilized a small number of ion channels and Ca^2+^-ATPases (Table 1) to maintain comparably low [Ca^2+^] in the cytoplasm. For example, both possess a single VGCC in Cch1 and its regulatory subunit Mid1 (budding yeast)/Yam8 (fission yeast). Both yeasts use a vacuolar Ca^2+^ pump Pmc1 and an ER/Golgi pump in Pmr1. In response to hypo-osmotic shocks, both yeasts trigger intracellular Ca^2+^ spikes and the CaN-Prz1 pathway [35,50,83]. CaM of both yeasts targets the pericentrin-like protein in SPBs during mitosis [60,84]. In both, CaN activates a Zinc finger transcription factor Crz1 (budding yeast)/Prz1 (fission yeast) through dephosphorylation under stress conditions [70,85].

One of the most striking differences between budding and fission yeast is in CaM. In budding yeast, Cmd1 can only bind three Ca^2+^ ions maximally. Its capacity for Ca^2+^-binding is not essential for budding yeast [86]. In contrast, fission yeast CaM can bind four Ca^2+^ ions simultaneously. Its ability to bind Ca^2+^ is indispensable for fission yeast viability [58]. Therefore, only fission yeast Cam1 needs Ca^2+^ for its essential functions.

There are other differences between these two yeasts (Table 1). Fission yeast possesses two homologues of the bacterial mechanosensitive ion channel MscS, Msy1 and Msy2, which are absent in budding yeast (Table 1). Although budding yeast uses a Transient Receptor Potential (TRP) channel Yvc1 to promote Ca^2+^ efflux at the vacuoles [87,88], no such vacuolar channel has been found in fission yeast yet. Budding yeast VGCCs, consisting of Mid1, Cch1, and Ecm7, are essential for Ca^2+^ signaling during mating [89,90]. The ER-localized putative Ca^2+^ channel Csg2 promotes autophagy in budding yeast cells [91], but no such ER Ca^2+^ channel has been identified yet in fission yeast. Budding yeast Ca^2+^ homeostasis has been examined using the genetically encoded Ca^2+^ reporters of either aequorin [92] or GCaMP [93]. Furthermore, a computation model has been constructed to simulate Ca^2+^ homeostasis in *S. cerevisiae* cells [94]. However, no cytokinetic Ca^2+^ spikes have yet been identified in this yeast.

**Table 1:** Comparison of Ca^2+^ homeostasis and signaling between two yeasts.

		*S. cerevisiae*	*S. pombe*
Ca^2+^ Channels	Voltage-gated	Cch1+Mid1+Ecm7 [24,95]	Cch1+Yam8 [50,96]
	TRP family	Yvc1 [87]	None identified
	Mechanosensitive Pkd2 family	Flc1, Flc2, Flc3, and YOR365C [83,97]	Pkd2, Trp1322, and Trp663 [44]
	Mechanosensitive MscS-like	None identified	Msy1 and Msy2 [47]
	Others	Csg2 [91]	None identified
Ca^2+^-ATPases	ER	Spf1 [98]	Pmr1 and Cta4 [27,28]
	Golgi	Pmr1 [21]	None identified
	Vacuolar	Pmc1 [99]	Pmc1 [27]
Ca^2+^-binding EF-hand proteins	Calmodulin	Cmd1 [100]	Cam1 [57]
	Calcineurin	Cna1/Cna2+Cnb1 [101]	Ppb1+Cnb1 [67,102]
	Centrin	Cdc31 [103]	Cdc31 [72]
	Myosin light chains	Mlc1 and Mlc2 [104,105]	Cdc4 and Rlc1 [73,74]
	Neuronal Ca^2+^ sensor	Frq1 [77]	Ncs1 [75]

## 8. Comparison Between Regulation of Cytokinesis by Ca^2+^ in Yeast and Animal Cells

The discovery of cytokinetic spikes in fission yeast cells naturally invites a comparison to the Ca^2+^ waves discovered earlier in animal cells. In both, cytokinesis is accompanied by two waves of Ca^2+^ transients (Table 2). In addition to amphibian or fish embryonic cells, mammalian embryonic cells also trigger Ca^2+^ waves during their cytokinesis [106]. In animal cells, the first wave of transients coincides with the start of cleavage furrow ingression. This so-called “Furrowing wave” originates at the equatorial division plane, exactly where the membrane starts to ingress [1,6]. It then spreads towards the cell pole at a rate of ~4 µm/min, a comparably slow one among Ca^2+^ waves [107]. The second wave of the transients accompanies the separation of daughter cells, the final step of cytokinesis [1,35]. It was thus aptly named the “Zipping wave” due to the zipping motion of the two separated cells. There are striking similarities between the temporal regulation of cytokinetic Ca^2+^ transients in yeast and that in animal cells.

Nevertheless, there are some critical differences between Ca^2+^ transients of yeast and those of animal cells (Table 2). First is their respective timescale. Yeast Ca^2+^ spikes, lasting up to three minutes, are much longer-lived than the Ca^2+^ transients discovered in embryonic cells, which have a lifetime of just a few seconds [1,4]. Second is their vastly different spatial distribution. Yeast Ca^2+^ spikes are global in their intracellular distribution [35]. In contrast, Ca^2+^ transients of the much bigger animal cells rise locally at the equatorial division plane, before spreading throughout the division cells. The spatial distribution of Ca^2+^ transients may be due to the different sizes of these two systems.

Animal and yeast cells also differ in their respective regulatory mechanism of these cytokinetic Ca^2+^ transients (Table 2). The Ca^2+^ influx of animal cells contributes only partially to the cytokinetic Ca^2+^ waves [108]. This is mediated through the store-operated Ca^2+^ entry (SOCE) system consisting of the plasma membrane-localized ORAI channel and its interacting partner STIM at the ER (for reviews, see [109,110]). Thus far, no SOCE has been found in yeast [111]. In comparison, yeast cells depend on the mechanosensitive channel Pkd2 to mediate Ca^2+^ influx during cytokinesis. Animal cells possess two polycystin homologues in PC1 and PC2. Like yeast Pkd2, the animal polycystin PC2 is a mechanosensitive channel [112,113]. However, the role of polycystins in animal cell cytokinesis remains unknown. The animal cells also mobilize intracellular stored Ca^2+^ from the ER for the transients. The ER Ca^2+^ channel inositol-1,4,5-triphosphate (IP_3_) receptor promotes cytokinetic Ca^2+^ transients [4,114]. In contrast, the role of Ca^2+^ efflux during yeast cytokinesis remains unknown.

**Table 2:** Comparison of cytokinetic Ca^2+^ transients between animal and yeast cells.

	*Animal*	*Yeast*
Cytokinetic Ca^2+^ transients	Furrowing wave Zipping wave [1]	Constriction spike Separation spike [35]
Efflux channel promoting Ca^2+^ transients	IP3 receptor [37]	None identified
Influx channel promoting Ca^2+^ transients	ORAI [108]	Pkd2 [46]
Potential targets of Ca^2+^ transients	Calmodulin [115]	Cam1 [58]
	Calcineurin [116]	Ppb1+Cnb1 [67,117]
	MLCK [118]	None identified
	CaMKII	Cmk1 and Cmk2
	Annexin [119]	None identified
	VAMP2 [120]	None identified

Animal and yeast cells also differ significantly on the targets of their Ca^2+^ cytokinetic transients (Table 2), although exocytosis is likely targeted by Ca^2+^ transients in both. Most animal cells including fruit flies utilize the Ca^2+^/camodulin-sensitive myosin light chain kinase (MLCK) to trigger actomyosin contractility during cytokinesis [118]. In comparison, yeast cells do not possess any MLCK homologue. In animal cells, CaM promotes the assembly of the central spindle, which is essential for animal cell cytokinesis [121]. CaMKII (Ca^2+^/calmodulin-dependent kinase II), a potential target of Ca^2+^ transients, activates the small GTPases RhoA and Cdc42 to re-organize the actin cytoskeleton during animal cell cytokinesis [115]. In comparison, yeast CaMKII homologues, Cmk1 and Cmk2, have no clear role in cytokinesis. In animal cells, Ca^2+^-sensitive proteins such as VAMP2 and annexin also contribute to cytokinesis [119,122], but their homologues in yeast cytokinesis have not been determined. Overall, many potential targets of cytokinetic Ca^2+^ transients remain to be identified in either fission yeast or animal cells.

## 9. Conclusions and Open Questions

Many exciting questions remain open in our understanding of the Ca^2+^ transients accompanying cytokinesis. Some of them require improved technology, while others demand more conceptual advances. Here, I will list a few of the most important ones.

The main challenge in determining the role of Ca^2+^ in yeast cytokinesis is our lack of understanding of Ca^2+^ homeostasis in fission yeast. To start, we do not know how fission yeast cells maintain cytoplasmic Ca^2+^ concentration. We have yet to determine whether Ca^2+^ efflux from either the ER or vacuoles contributes to such homeostasis. Adding to the mystery is that no ER- or vacuole-specific efflux channels have been identified in fission yeast. Organelle-specific Ca^2+^ reporters will help us to measure Ca^2+^ efflux in yeast cells.

Another challenge is to identify the ion channels required for cytokinetic Ca^2+^ spikes. To our knowledge, Pkd2 is the only known channel that plays a clear role in promoting cytokinetic Ca^2+^ transients. It remains to be determined whether any other yeast channels, such as the putative voltage-gated channel Cch1 and the putative mechanosensitive channels Msy1 and Msy2 [47,48], contribute to the spikes. More work is also needed to identify novel fission yeast ion channels through a combination of yeast genetics, Ca^2+^ imaging, and electrophysiology.

Several questions remain unanswered about the molecular role of Pkd2 in cytokinesis. First, it remains unknown whether Pkd2 is a non-selective cation channel. The human homologue PC2 is permissive to both K^+^ and Na^+^, in addition to Ca^2+^ [123]. Secondly, the atomic structure of the Pkd2 channel, likely in an oligomeric form, remains largely unknown, despite the monomeric structure predicted by AlphaFold [44]. The human Pkd2 homologues PC-1 and PC-2 can form either a homo-tetramer or a hetero-tetramer channel [124,125]. Lastly, it remains to be determined whether Pkd2 does more than promote Ca^2+^ spikes during cell separation. The cytokinetic defects of *pkd2* mutant cells differ significantly from those cells whose Ca^2+^ spikes have been largely blocked. In particular, *pkd2* mutant cells take much longer than wild-type cells to separate. In comparison, cells treated with the Ca^2+^ chelator EGTA separate normally, but often lyse following the separation. Further studies would be needed to identify the signaling pathways that Pkd2 interacts with during cytokinesis.

The interplay between Ca^2+^ spikes and the re-organization of actin cytoskeletal structures during cytokinesis has yet to be explored. This is an exciting direction in light of the recent finding that Pkd2 modulates the assembly of the actomyosin contractile ring [126]. Hypermorphic *pkd2* mutation increases the number of both actin filaments and type II myosins in the ring. Of course, there is also the question of whether Ca^2+^ spikes can activate myosins during cytokinesis. In animal cells, Ca^2+^ can activate the myosin light chain kinase (MLCK) to phosphorylate the type II myosin regulatory light chain. This activates myosin II and triggers contraction of the ring. However, fission yeast does not possess an MLCK homologue. This makes it unlikely that Ca^2+^ spikes can directly activate the type II myosins Myo2 and Myp2. Moving forward, more studies will be needed to determine whether Ca^2+^ spikes can activate other myosins such as type I and V myosins during cytokinesis.

Another interesting question to answer is whether there are any connections between cytokinetic Ca^2+^ transients and intracellular bioelectricity. Bioelectricity refers to signaling due to a change in the plasma membrane potential (for a review, see [127]). The resting potential of the plasma membrane usually remains negative. Either hyper- or hypo-polarization of the membrane can change the potential, resulting in activation of voltage-sensitive ion channels and ion pumps. Cytokinesis could trigger intracellular bioelectricity, leading to cytokinetic Ca^2+^ transients. Future studies will be needed to measure plasma membrane potential throughout cytokinesis of fission yeast cells.

The discovery of cytokinetic Ca^2+^ transients in the unicellular model organism fission yeast presents a new opportunity to determine the role of Ca^2+^ during cytokinesis. With a combination of quantitative fluorescence microscopy, yeast genetics, and in vitro biochemistry, we may finally be able to tackle the decades-old questions of where Ca^2+^ transients come from and what their roles are during cytokinesis.

## Data Availability

No new data were created or analyzed in this study.
